# Supplementary figures and images for: Maintaining immunogenicity of blood stage and sexual stage subunit malaria vaccines when formulated in combination
Source: PLoS One. 2020 Apr 29;15(4):e0232355. doi: 10.1371/journal.pone.0232355 (PMC7190115; doi:10.1371/journal.pone.0232355)

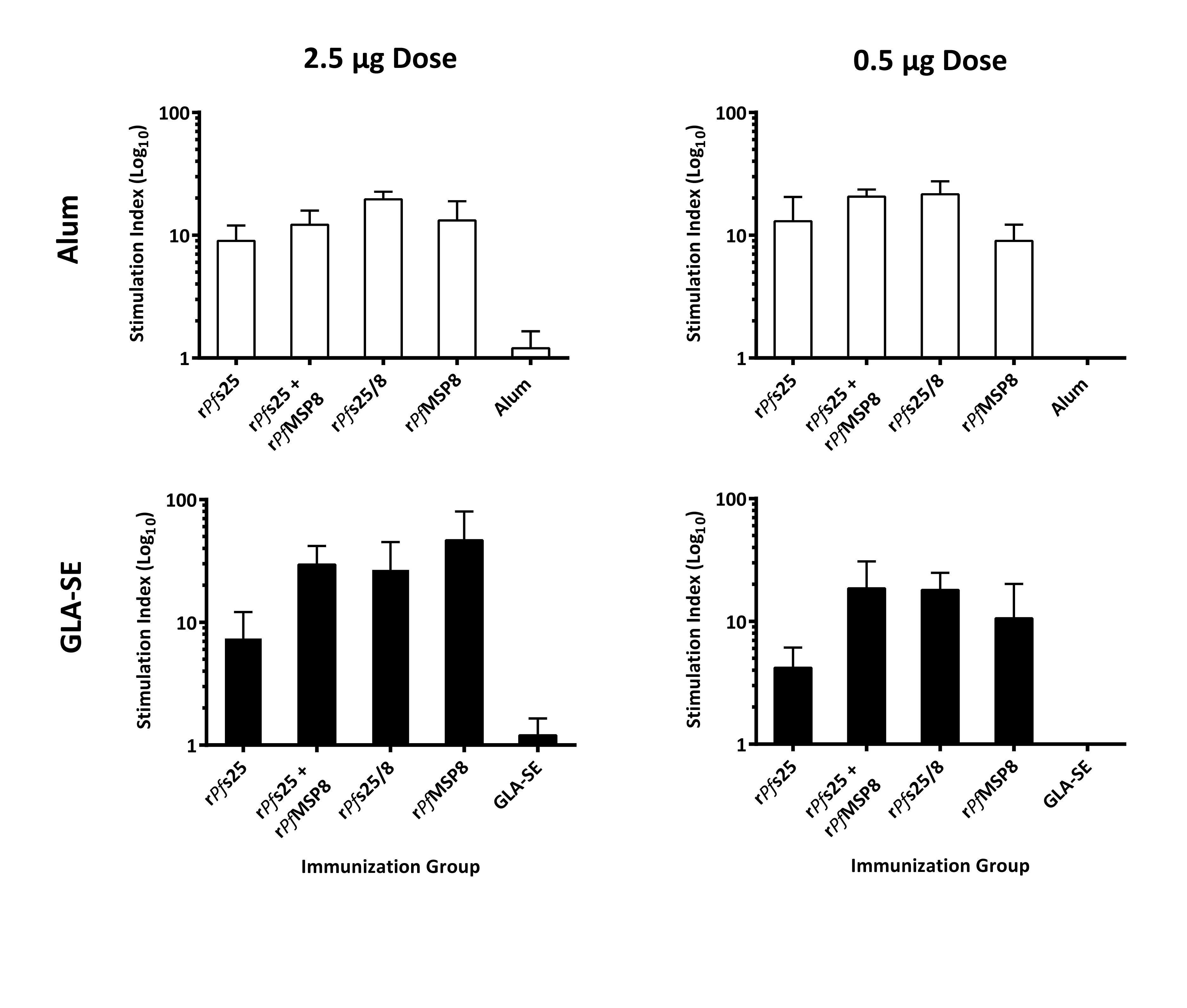


**Figure S1**

Supplement: S1 Fig — CB6F1/J splenocytes (2 x 10^5^/ well) harvested from the indicated immunization groups were stimulated ex vivo in triplicate with rPfs25/8 (2 μg/ well) for 96 hrs. [3H]-thymidine (1 μCi/ well) was added for the final 18 hours. Average counts of incorporated [3H]-thymidine were measured for rPfs25/8-stimulated wells and converted into a Stimulation Index (SI) that represents the fold change in proliferation of the indicated condition over the corresponding control wells (media alone). Graphs depict mean SI +/- standard deviation. (DOCX) [file pone.0232355.s001.docx]

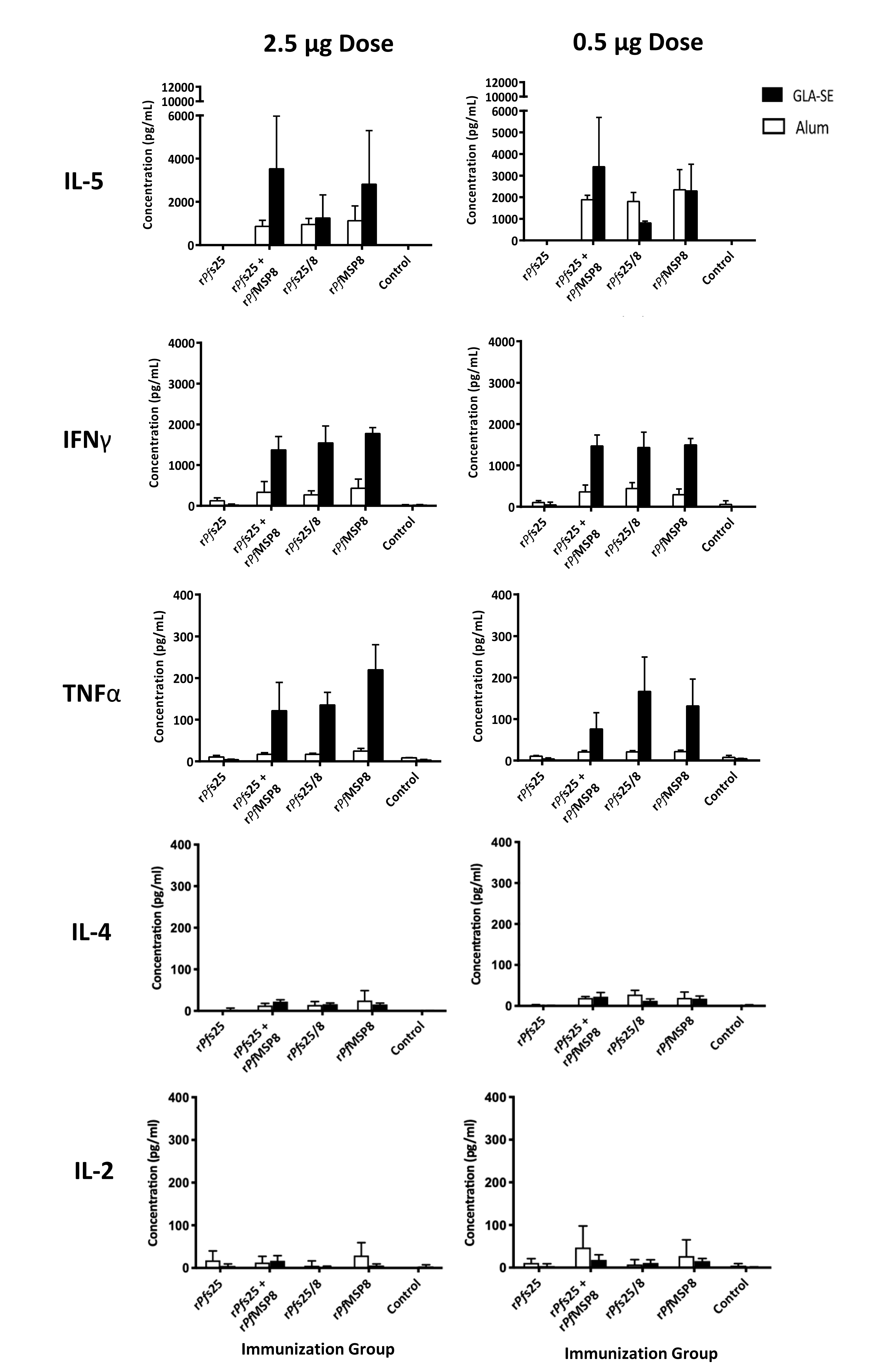


**Figure S2**

Supplement: S2 Fig — CB6F1/J splenocytes (5 x 10^5^/well) were harvested from the indicated immunization groups and stimulated ex vivo in triplicate with rPfMSP8 (2 μg/well) or in media alone for 96 hours. Culture supernatants were collected and analyzed for production of IL-5, IFNγ, TNFα, -4 and IL-2 using a multiplex assay (Luminex®). To calculate the final concentration of each analyte, the levels found in the corresponding unstimulated conditions were subtracted out as background. Graphs depict mean concentration of each analyte +/- standard deviation. (DOCX) [file pone.0232355.s002.docx]

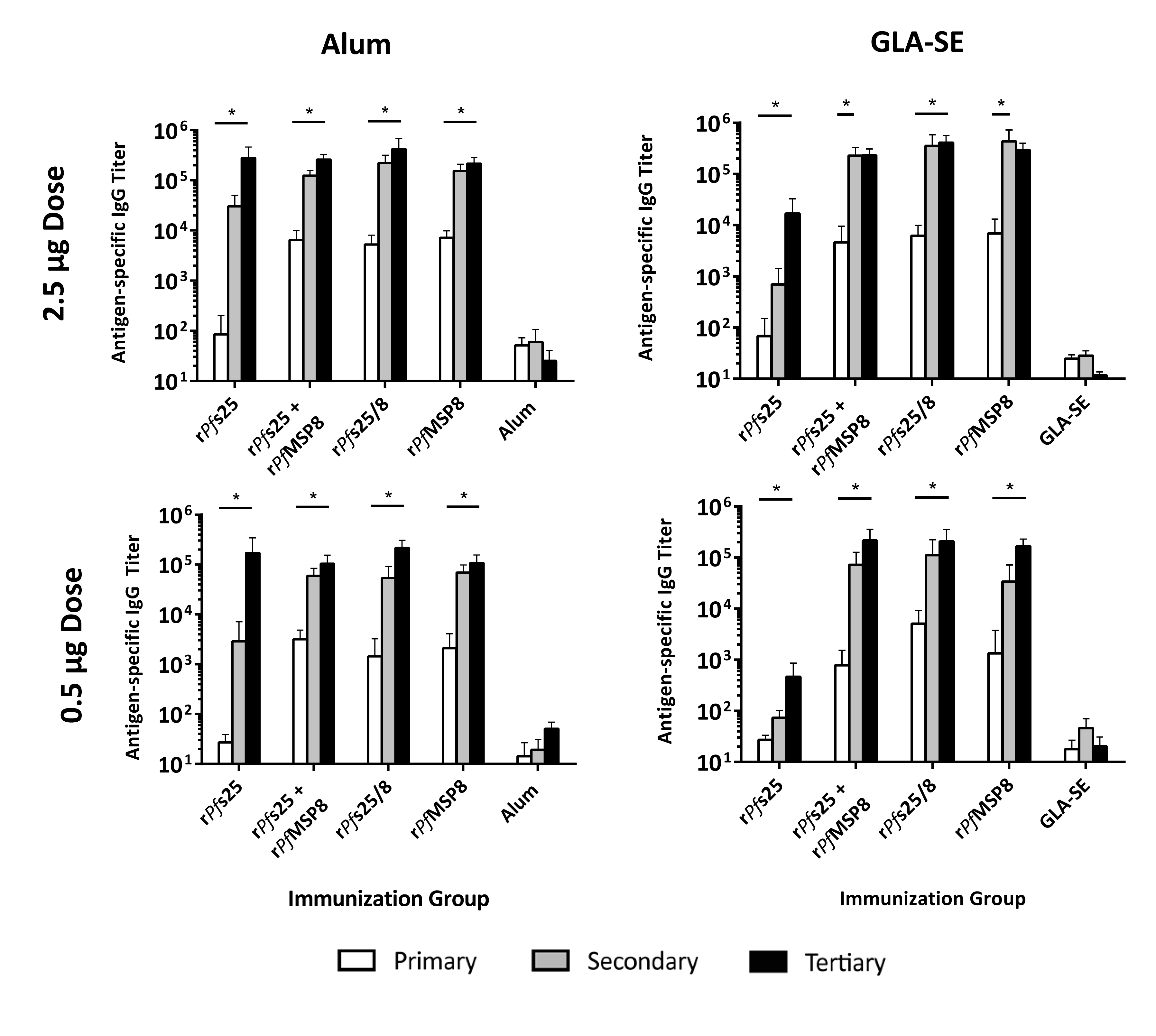


**Figure S3**

Supplement: S3 Fig — CB6F1/J sera collected 3 weeks following each subcutaneous immunization were analyzed for antigen-specific IgG via ELISA using plates coated with rPfs25/8 (0.25 μg/well). Graphs depict mean IgG titers +/- standard deviation. Asterisks over bars within groups indicate significant boosting of antigen-specific responses over time (Friedman Test; P < 0.05 considered significant). (DOCX) [file pone.0232355.s003.docx]

**
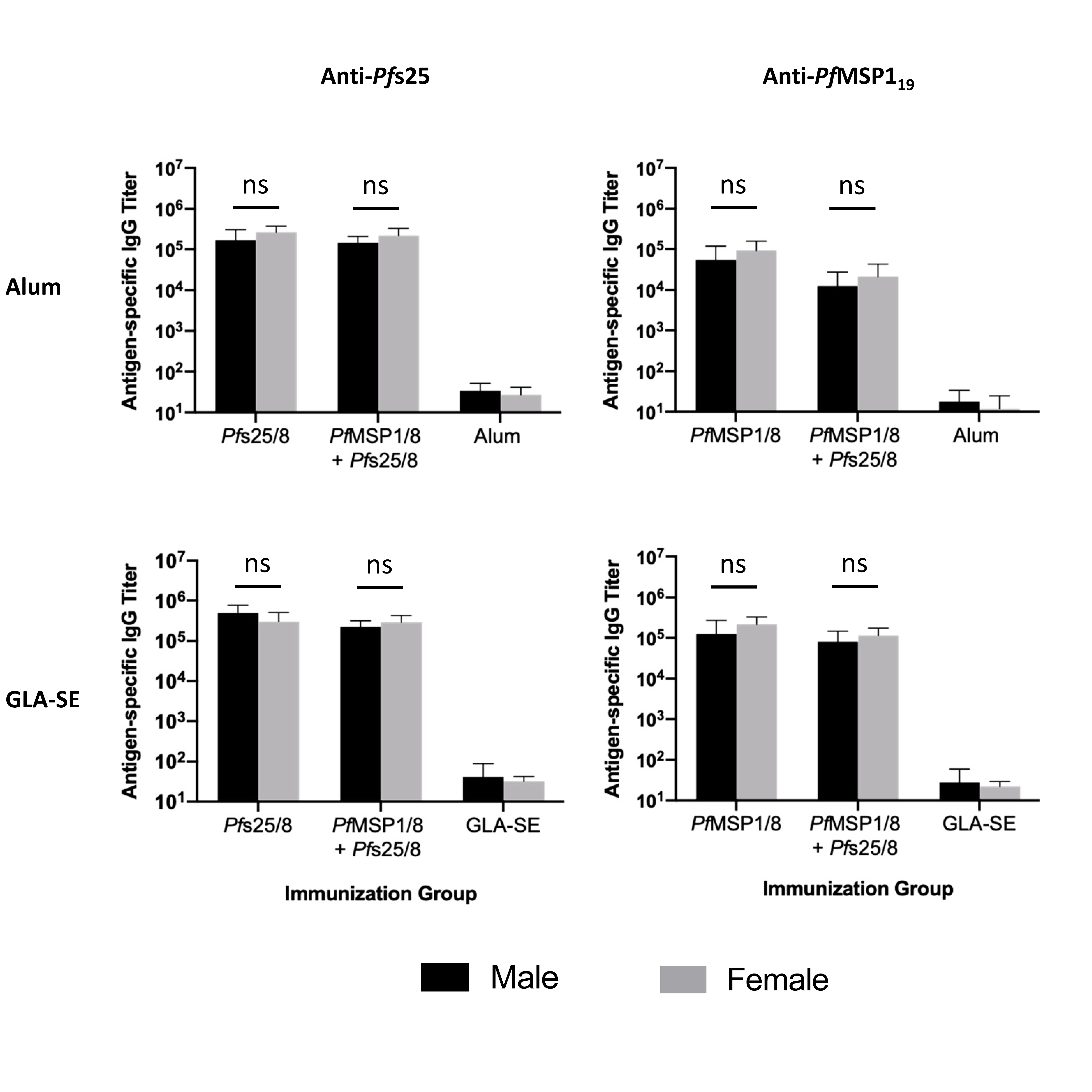
**

**Figure S4**

Supplement: S4 Fig — CD1 mice (10/group with 5 male and 5 female mice) were immunized as indicated and sera were collected following the third immunization. Titers of antigen-specific IgG were measured by ELISA with plates coated with rPfs25 or rGST-PfMSP119 (0.25 ug/well). Graph depicts mean IgG titers +/- standard deviation. Antibody responses in male and female mice within the same immunization group were compared. Statistical significant of differences between sexes were evaluated (Mann-Whitney U Test; P < 0.05 considered significant; ns, not significant). (DOCX) [file pone.0232355.s004.docx]

**
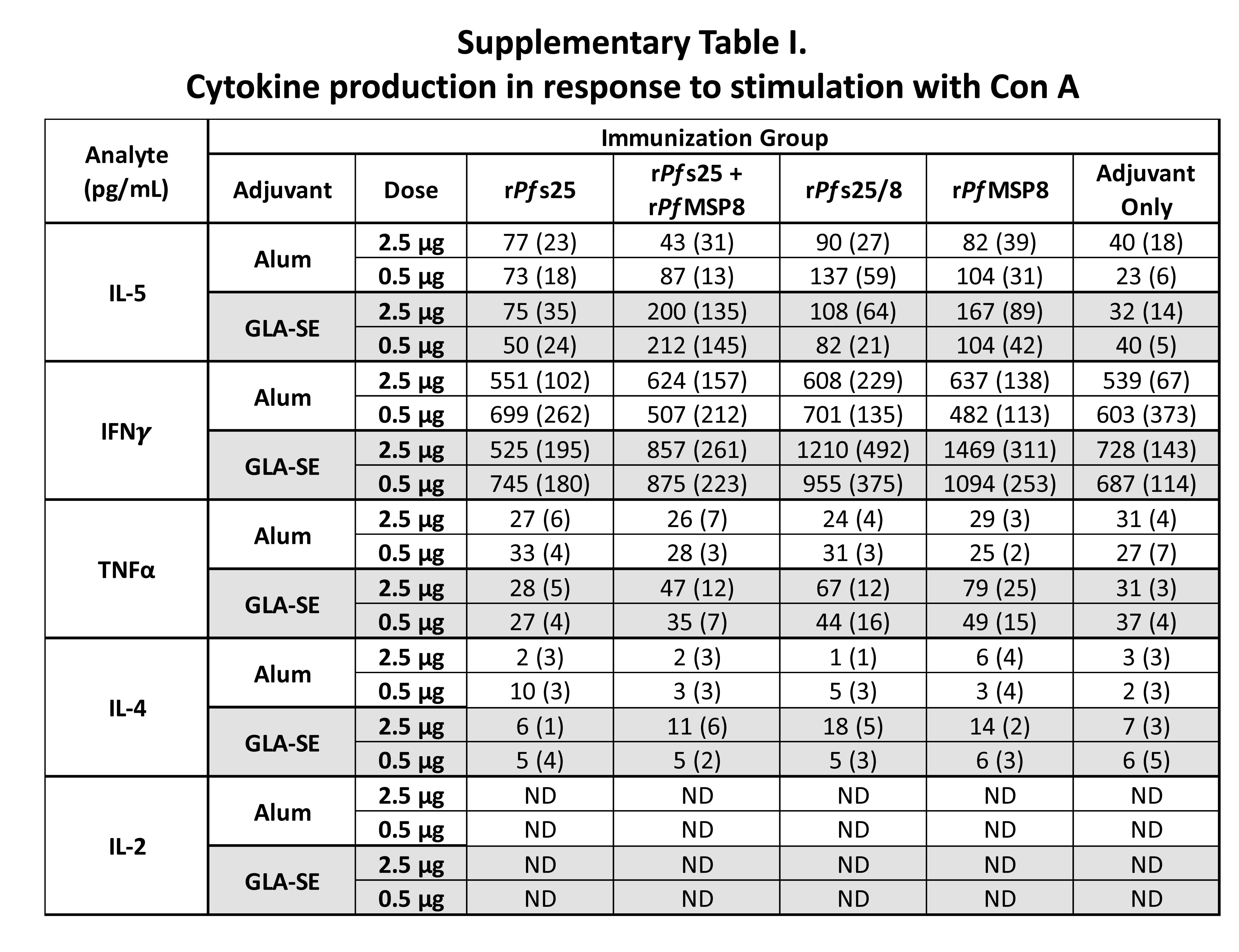
**

Supplement: S1 Table — Splenocytes (5 x 10^5^/well) were harvested from groups of CB6F1/J mice (n = 5) immunized as indicated and stimulated ex vivo with Con A (0.2 μg/well) or cultured in media alone for 96 hours. Culture supernatants were collected and analyzed for production of IL-5, IFNγ, TNFα, IL-2 and IL-4 using a multiplex assay (Luminex®). To calculate the final concentration of each analyte, the levels found in the corresponding unstimulated conditions were subtracted as background. (ND = not detected). (DOCX) [file pone.0232355.s005.docx]

**
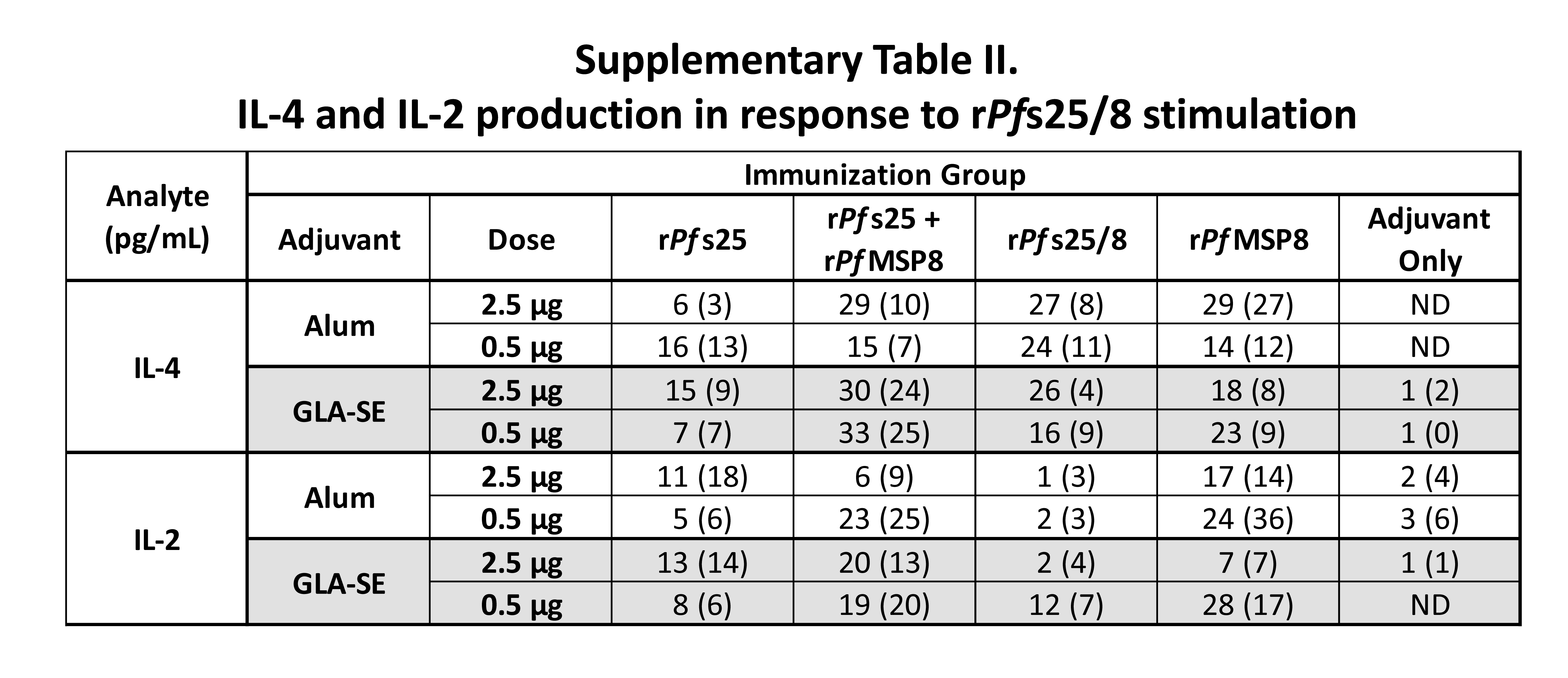
**

Supplement: S2 Table — Splenocytes (5 x 10^5^/well) were harvested from groups of CB6F1/J mice (n = 5) immunized as indicated and stimulated with ex vivo with rPfs25/8 (2 μg/well) or cultured in media alone for 96 hours. Culture supernatants were collected and analyzed for production of IL-2 and IL-4 using a multiplex assay (Luminex®). To calculate the final concentration of each analyte, the levels found in the corresponding unstimulated conditions were subtracted as background. (ND = not detected). (DOCX) [file pone.0232355.s006.docx]
